# Foreign Summary

**Published:** 1838-06-06

**Authors:** 


### FOREIGN SUMMARY.

*On the use of Chloride of Lime in Wounds attended with much pain, by Dr. Chopin.*—In wounds produced by contusion, laceration, or by the explosion of gunpowder, where there is much pain, speedy and certain relief, says Dr. C., is produced by chloride of lime. That this relief is not the effect of cold or any other cause than the chloride in solution, the author is convinced by many experiments. Charpie, moistened with the same solution, has been also found a useful application in relieving the pains which sometimes follow delivery, which depend on small excrescences in the vagina. That such is frequently the case, Dr. C. is convinced from repeated examination. Excoriated breasts are most efficiently treated by the use of the same external application.—*B. and F. Med. Rev. from Jahrbücher der in-und-ausländischen gesammten Medicin.* Heft i. 1837.

*Extirpation of the Uterus.*—MM. Capuron and Lisfranc lately read to the Academy their report on the following case. Professor Laserre was consulted by a middle-aged woman for an inversion and prolapsus of the womb, which had been induced by the violent traction employed by a midwife at her last accouchement, two years before. From that period, she had suffered repeatedly from alarming uterine hæmorrhage.

The tumour was of a pyriform shape, irregular and knobby on its surface, and the narrowest part or pedicle was girt with a hard ring (bourrelet) in the situation of the uterine orifice. Finding that it was quite impracticable to replace the uterus, M. Laserre determined to extirpate it, by means of a ligature passed round its pedicle, and tightened as firmly as the patient could bear. The acute pain induced by the ligature was calmed by opiates. 'Apres quelque temps' a second ligature was applied more tightly; but this operation caused such severe suffering, that it was found necessary to remove the string. The attempt was subsequently made several times; but the same alarming symptoms always coming on, the surgeon was obliged to resort to another practice. Drawing the tumour out, as far as he could, and finding that part only of the pedicle had been destroyed by the ligature, he passed another ligature tightly round it, and then removed the mass with one stroke of the knife. Peritonitis supervened; but a rigorous antiphlogistic practice speedily dissipated all the unpleasant symptoms. At the end of the fifth day 'l'etat de la malade etait satisfaisant.' On the following week, a painful swelling of the left thigh and leg came on; but this also gradually subsided, and in the course of five weeks from the date of the operation, the cure was complete. When the case was laid before the Academy last June, a year had elapsed since the period of cure. During that time, there had been no appearance of catamenia, and not even the 'prodromes' of the discharge had been experienced; whence M. Laserre concludes that this secretion must proceed altogether from the uterus, and not from the vagina. The patient has been able, we are informed, 'se livrer au coit comme avant, et elle y approuve la meme jouissance et les memes sensations.'

M. Capuron, in his report to the Academy on the preceding case, expressed his conviction that the uterus had been really extirpated; but adds, that it would have been more satisfactory if the details of the examination, and of the characters of the prolapsed tumour, had been more minutely and explicitly given. With respect to the absence of the catamenia, M. Laserre has been rather precipitate in his conclusion that they will never return in future; and he appears not to be aware that in several of the recorded cases of extirpated uterus, the menstrual discharge has been renewed, after having been absent for several years.

M. Nacquart directed the attention of the members to a colored engraving, intended to represent the oozing of the menstrual discharge from the mucous surface of the vagina, in a thesis published by Osiander.—*Archives Generales, from Medico-Chirurg. Review.*

*Treatment of Burns, by M. Velpeau, with Diachylon Plaster.*—The ever active surgeon of the La Charité recommends the employment of strips of diachylon plaster to burned surfaces, in preference to the ordinary applications, such as cold water, solution of the chlorides, Carron oil, &c. The advantage which it possesses over these means, according to M. V., is its being well adapted for all the various stages or degrees of the injury—from the simple irritation of the skin, caused by scalding, to the complete destruction and mortification of it and the subjacent cellular tissue. He recommends that the strips of the plaster be renewed every two or three days, and assures us that burns 'du premier degré guerissent immediatement;' those of the second degree in four or six days; those of the third degree in from eight to fifteen days; and those of the fourth degree in from fifteen to thirty days.

When the injury is attended with the destruction of the parts, and is therefore to be cured by ulceration and granulation, the process of cicatrization goes on under the plaster in several points at the same time, and not merely 'de proche en proche,' or from the circumference to the centre; as is ordinarily the case under the common dressings.—*Ib.*

